# Gender differences in hereditary hemorrhagic telangiectasia severity

**DOI:** 10.1186/s13023-020-1337-5

**Published:** 2020-03-02

**Authors:** J. M. Mora-Luján, A. Iriarte, E. Alba, M. A. Sánchez-Corral, P. Cerdà, F. Cruellas, Q. Ordi, X. Corbella, J. Ribas, J. Castellote, A. Riera-Mestre

**Affiliations:** 1 HHT Unit, Hospital Universitari de Bellvitge, C/Feixa Llarga s/n. L’Hospitalet de Llobregat, 08907 Barcelona, Spain; 2 Internal Medicine Department, Hospital Universitari Bellvitge, Barcelona, Spain; 3 Bellvitge Biomedical Research Institute (IDIBELL), Barcelona, Spain; 4 Radiology Department, Hospital Universitari Bellvitge, Barcelona, Spain; 5 Cardiology Department, Hospital Universitari Bellvitge, Barcelona, Spain; 6 Otorhinolaryngology Department, Hospital Universitari Bellvitge, Barcelona, Spain; 7 Faculty of Medicine and Health Sciences, Universitat Internacional de Catalunya, Barcelona, Spain; 8 Pneumology Department, Hospital Universitari Bellvitge, Barcelona, Spain; 9 Liver Transplant Unit, Department of Digestive Diseases, Hospital Universitari Bellvitge, Barcelona, Spain; 10 Faculty of Medicine and Health Sciences, Universitat de Barcelona, Barcelona, Spain

**Keywords:** Hereditary hemorrhagic telangiectasia, Gender, Arteriovenous malformations, Rare diseases

## Abstract

**Background:**

Gender differences in organ involvement and clinical severity have been poorly described in hereditary hemorrhagic telangiectasia (HHT). The aim of this study was to describe differences in the severity of HHT manifestations according to gender.

**Methods:**

Severity was measured according to Epistaxis Severity Score (ESS), Simple Clinical Scoring Index for hepatic involvement, a general HHT-score, needing for invasive treatment (pulmonary or brain arteriovenous malformations -AVMs- embolization, liver transplantation or Young’s surgery) or the presence of adverse outcomes (severe anemia, emergency department -ED- or hospital admissions and mortality).

**Results:**

One hundred forty-two (58.7%) women and 100 (41.3%) men were included with a mean age of 48.9 ± 16.6 and 49 ± 16.5 years, respectively. Women presented hepatic manifestations (7.1% vs 0%) and hepatic involvement (59.8% vs 47%), hepatic AVMs (28.2% vs 13%) and bile duct dilatation (4.9% vs 0%) at abdominal CT, and pulmonary AVMs at thoracic CT (35.2% vs 23%) more often than men. The Simple Clinical Scoring Index was higher in women (3.38 ± 1.2 vs 2.03 ± 1.2), and more men were considered at low risk of harboring clinically significant liver disease than women (61% vs 25.3%). These differences were mantained when considering HHT1 and HHT2 patients separetely. Duodenal telangiectasia were more frequent in men than women (21% vs 9.8%). Invasive treatments were more frequently needed in women (28.2% vs 16%) but men needed attention at the ED more often than women (48% vs 28.2%), with no differences in ESS, HHT-score, anemia hospital admissions or mortality.

**Conclusions:**

HHT women showed more severe hepatic involvement than men, also among HHT1 and HHT2 patients. Women had higher prevalence of pulmonary AVMs and needed invasive procedures more frequently, while men needed attention at the ED more often. These data might help physicians to individualize HHT patients follow-up.

## Introduction

Hereditary hemorrhagic telangiectasia (HHT) or Rendu-Osler-Weber syndrome (ORPHA774) is a rare autosomal-dominant vascular disease characterized by telangiectases and larger vascular malformations (VMs) [1, 2]. Mutations in endoglin (*ENG*) and activin A receptor type II-like 1 (*ACVRL1*) genes are detected in approximately 90% of cases submitted to molecular diagnosis for clinical suspicion of HHT and cause HHT1 or HHT2, respectively [3–6]. Mutations in *SMAD4* (encoding the transcription factor Smad4) have been described in less than 2% of the HHT population and cause juvenile polyposis/HHT overlap syndrome [3]. Endoglin (encoded by *ENG*) is an auxiliary co-receptor at the endothelial cell surface that promotes BMP9 signalling through the activin receptor-like kinase 1 (ALK1; encoded by *ACVRL1*). Both proteins contribute to the signalling hub formed by BMP9-Endoglin-ALK1-Smad with high impact in angiogenesis [7].

HHT can be diagnosed either through molecular genetic test or using the Curaçao clinical criteria (recurrent epistaxis, muco-cutaneous telangiectasia, visceral lesions and family history) [8–10]. Telangiectasia is the hallmark in HHT and shows dilated post capillary venules directly connected with dilated arterioles losing the capillary bed [11]. Telangiectasia in nasal mucosae cause spontaneous and recurrent epistaxis which is the most common and usually the earliest clinical manifestation of HHT [4, 6, 12, 13]. Pulmonary arteriovenous malformations (AVMs) and brain VMs are more common in patients with HHT1 and vascular hepatic malformations in HHT2 patients [4, 8, 14–17]. Despite these phenotype patterns, there is significant intra-familial as well as inter-familial clinical variability among HHT patients [18]. Screening for possible VMs is recommended for early detection and appropiate treatment [10, 19, 20].

Although the offspring of HHT patients have a 50% chance of inheriting a mutation, some studies have found a higher prevalence of HHT in women compared with men [12, 21, 22]. One possible explanation for this observation is that women have significantly higher rates of consultation with primary care providers, but the existence of other biological reasons is unknown [23]. Moreover, the evidence of gender influence in HHT severity is scarce and not directly addressed [10, 12, 24, 25]. Data from the largest series of HHT patients with liver transplantation from the European Liver Transplant Registry, showed that 35 (87.5%) out of 40 patients were women [24]. Actually, female gender was considered as a risk factor in a multivariate analysis when defining a score for clinically significant hepatic involvement in HHT [19]. Nonetheless, gender was not found to be associated with adverse outcomes in a series of 393 patients that defined a severity score for HHT [25]. However, most of these studies focused on isolated organ involvement and were not specifically designed to analyze gender differences. In the present study, we aimed to assess how gender influence the severity of HHT.

## Material and methods

### Study design

This is a prospective non-interventional study including all consecutive patients visited in a HHT multidisciplinary referral unit in a university hospital. This HHT Unit serves adult patients from all over Catalonia (Spain), which is about 7.5 million inhabitants. The study period was September 2011 to January 2019. Patients with a “definite” diagnosis according to the Curaçao Criteria (meet ≥3 criteria) or a positive genetic study, were included. Patients who met < 3 Curaçao Criteria without positive genetic test, and those in whom screening was not completed, were excluded [8–10]. Oral informed consent was obtained from all participants. The study was approved by the Clinical Research Ethics Committee of the Hospital Universitari de Bellvitge. We followed the Strengthening the Reporting of Observational Studies in Epidemiology (STROBE) statement guidelines for observational cohort studies [26].

The aim of the study was to assess differences in clinical severity according to gender in a large series of patients with objectively confirmed HHT. A secondary objective was to analyze gender differences in severity among patients with HHT1 or HHT2, separately.

### Screening for vascular involvement

Clinical characteristics at baseline and complementary tests were prospectively collected. Screening for vascular involvement was done according to guidelines and expert’s recommendations [9, 10, 27]. All patients were examined by an ear, nose and throat (ENT) physician expert on HHT. Anemia was defined as hemoglobin levels < 12 g/dL in women and < 13 g/dL in men. Iron deficiency was defined as blood ferritin levels < 15 μg/L.

Hepatic manifestations were defined as clinical ischemic cholangitis or right-upper abdominal pain with bile duct dilation, signs and symptoms of high-output cardiac failure secondary to hepatic involvement, or hepatic encephalopathy in patients with objectively confirmed hepatic VMs [10, 28]. Pulmonary manifestations in patients with pulmonary AVMs were defined as the presence of respiratory failure (PaO2 < 60 mmHg) without any other etiologic cause, hemoptysis, stroke or transient ischemic attack without cardiac arrhythmia or associated myocardiopathy, or brain abscess.

In order to calculate cardiac index (L/min/m^2^) and for the screening of pulmonary visceral involvement, a contrast transthoracic echocardiography (TTE) was performed [10, 27]. The Barzilai scale was used to establish the degree of right-left (R-L) shunt [29]. All patients with R-L shunt Grade ≥ 2 and those with previous pulmonary AVMs embolitzation underwent a thoracic computed tomography (CT) to objectively confirm the presence of pulmonary AVMs. In addition, an abdominal CT was performed to study hepatic and/or other abdominal AVMs. Hepatic involvement at CT was defined according to the presence of telangiectasia, tortuous or enlarged hepatic artery (> 6 mm diameter), any of the three classical patterns of HHT vascular shunts (portovenous, arteriovenous or arterioportal) or regenerative or nodal focular hyperplasia [10, 28]. Other intraabdominal vascular involvement rather than hepatic, were also recorded.

Neurological involvement studies were carried out in cases of neurological symptoms or family history by a cerebral CT and/or magnetic resonance imaging (MRI) [10]. A gastrointestinal (GI) endoscopic digestive study was performed when there was disproportionate anemia to the degree of epistaxis or objectively confirmed overt GI bleeding [10, 27].

### HHT severity assessment

A clinical follow-up was carried out according to each of patient’s needs. HHT clinical severity was assessed using the following five items: Epistaxis Severity Score (ESS), Simple Clinical Scoring Index for clinically significant hepatic involvement, HHT-score, the need for invasive treatment or the presence of adverse outcomes related to HHT [19, 25, 30].

The ESS is an on-line tool that quantifies the severity of epistaxis according to different parameters occurring within the previous three months [30]. The ESS ranges from 0 to 10, defining epistaxis as mild (ESS 1–4), moderate (ESS 4–7) or severe (ESS ≥7). For each patient, baseline ESS and mean ESS assessed at each visit during follow-up were registered.

Liver involvement severity was determined by the Simple Clinical Scoring Index [19]. This index include four simple variables: age, gender, hemoglobin and serum alkaline phosphatase. The score ranges from 0 to 9 and stratifies patients with a value score < 3 to be at low-risk (< 5% probability), 3–5 at intermediate risk (5–80% probability) and ≥ 6 at high-risk (> 80% probability) of harboring clinically significant liver disease.

The HHT-score was calculated based on: chronic bleeding (maximum 2 points), presence of AVMs (maximum 3 points) and severe organ involvement (maximum 2 points) [25]. Patients were categorized as having mild (0–2), moderate (3–4) or severe (5–7) HHT disease.

Specific invasive treatment strategies were defined as pulmonary or brain VM’s embolization, liver transplantation or Young’s surgery [31]. Emergency Department (ED) assistance, hospital admission, severe anemia and overall mortality during follow-up were considered as adverse outcomes. Severe anemia was defined as hemoglobin level < 8 g/dL or the need for red blood cell (RBC) transfusion.

### Statistical analysis

A descriptive statistical analysis was performed for all categorical and continuous variables and expressed as proportions or means with standard deviations (SD), respectively. We used chi-square or Fisher’s exact tests to compare categorical data between groups. Continuous variables were compared using Student t test. Hazard ratios (HR) and corresponding 95% confidence intervals (CI) were calculated. We used two-tailed unpaired t-tests to compare normally distributed continuous data between two groups, and we used the Mann-Whitney U test for non-normally distributed continuous data comparisons. *P* values of < 0.05 were considered statistically significant. Analyses were performed using SPSS, version 18 for the PC (SPSS, Inc. Chicago, IL, USA).

## Results

### Baseline characteristics

During the study period, 290 patients attended our HHT multidisciplinary referral unit. Among them, 48 (16.5%) patients were excluded, 26 patients met < 3 Curaçao Criteria without a positive genetic test and 22 because screening was not completed. Finally, 242 patients were included, 142 (58.7%) women and 100 (41.3%) men with a mean age of 48.9 ± 16.6 and 49 ± 16.5 years, respectively. Women were less likely to have previous/current tobacco (28.9% vs 62%) or alcohol (4.2% vs 24%) use and diabetes (5.6% vs 14%) than men.

At diagnosis, no gender differences were found by the Curaçao Criteria. The genetic study was positive for *ENG* mutations in 80 (49 women) patients, for *ACVRL1* in 75 (42 women) and negative in 17 (11 women) patients (all of them with a “definite” diagnosis according to Curaçao Criteria). There were no statistically significant differences regarding the presence of anemia, iron defficiency or liver function alterations (Table 1).

**Table 1:** Clinical characteristics according to gender

	Female *N* = 142	Male *N* = 100	*P* value
Clinical characteristics,			
Age, years (mean ± SD)	48.9 ± 16.6	49 ± 16.5	0.995
Smoke (active or history)	41 (28.9%)	62 (62%)	< 0.001
Alcohol (active or history)	6 (4.2%)	24 (24%)	< 0.001
Hypertension	34 (23.9%)	25 (25%)	0.851
Diabetes Mellitus	8 (5.6%)	14 (14%)	0.026
Dyslipidemia	38 (26.8%)	29 (29%)	0.701
Heart failure*	5 (3.5%)	2 (2%)	0.703
Lung disease	20 (14.1%)	14 (14%)	0.985
Cancer	11 (7.7%)	11 (11%)	0.416
Venous thromboembolic disease	2 (1.4%)	4 (4%)	0.240
Atrial fibrillation	15 (10.6%)	4 (4%)	0.056
Curaçao criteria,			
Epistaxis	134 (94.4%)	99 (99%)	0.085
Telangiectasia	135 (95.1%)	99 (99%)	0.145
Visceral involvement	119 (83.8%)	73 (73%)	0.056
Family history	137 (96.5%)	91 (91%)	0.072
Curaçao criteria ≥3	135 (95.1%)	97 (97%)	0.315
Genetic test,	102 (71.8%)	70 (70%)	0.757
ENG	49 (34.5%)	31 (31%)	0.670
ACVRL1	42 (29.6%)	33 (33%)	0.407
Negative	11 (7.7%)	6 (6%)	0.515
Blood test,			
Hemoglobin levels, g/dL (mean ± SD)	12.5 ± 2.2	13.4 ± 2.9	0.007
Anemia**	45 (31.7%)	39 (39%)	0.247
Iron deficiency***	85 (59.8%)	59 (59%)	0.937
AST, μkat/L (mean ± SD)	0.35 ± 0.19	0.35 ± 0.12	0.874
AF, μkat/L (mean ± SD)	1.36 ± 0.84	1.97 ± 7.82	0.455
Bilirubin, μmol/L (mean ± SD)	8.67 ± 5.74	10.01 ± 7.10	0.133

Abbreviations: *ACVRL1* activin A receptor type II-like 1, *ENG* Endoglin, *AST* aspartate aminotransferase, *AF* alkaline phosphatase

* Heart failure due to cardiovascular heart disease

** Anemia was defined as hemoglobin levels < 12 g/dL in women and < 13 g/dL in men

*** Iron deficiency was defined as blood ferritin levels < 15 μg/L.

### HHT involvement

Predefined hepatic manifestations were more frequent in women than men (7.1% vs 0%). Women showed more often ischemic cholangitis or right-upper abdominal pain with bile duct dilation (4.2% vs 0%) and high-output cardiac failure caused by liver involvement (5.6% vs 0%), with resulting higher mean cardiac index (3.21 ± 0.87 vs 2.87 ± 0.75, L/min/m^2^). Hepatic involvement at CT was also more frequently found in women (59.8% vs 47%), mainly for arteriovenous shunt (28.2% vs 13%) and bile duct dilatation (4.9% vs 0%). There were no statistically significant differences in non-hepatic intra-abdominal HHT-related vascular involvement between genders.

R-L shunting grades ≥2 at contrast TTE were more frequently found in women compared to men (37.3% vs. 24%). This finding was consistent with a higher prevalence of pulmonary AVMs (35.2% vs 23%) at thoracic CT in women. Central nervous system manifestations or vascular imaging did not show significant differences beetwen genders. Overall, no differences were found in GI involvement between women and men (20.4% vs 23%). However, duodenal telangiectasia were more frequent in men than women (21% vs 9.8%) (Table 2).

**Table 2:** Gender differences in HHT involvement

	Female (n = 142)	Male (n = 100)	*P* value
***Hepatic assessment***			
*Hepatic manifestations*	10 (7.1%)	0	0.006
Ischemic cholangitis/right-upper abdominal pain	6 (4.2%)	0	0.044
Heart failure*	8 (5.6%)	0	0.022
Hepatic encephalopathy	0	0	–
*TTE*	139 (97.9%)	97 (97%)	0.693
Cardiac index, L/min/m^2^ (mean ± SD)	3.21 ± 0.87	2.87 ± 0.75	0.010
Cardiac Index > 4	17 (11.9%)	4 (4%)	0.055
*Abdominal CT*	126 (88.7%)	90 (90%)	0.754
*Hepatic involvement*	85 (59.8%)	47 (47%)	0.024
Arteriovenous shunt	40 (28.2%)	13 (13%)	0.026
Bile duct dilatation	7 (4.9%)	0	0.043
Portovenous shunt	13 (9.1%)	9 (9%)	0.590
Arterioportal shunt	28 (19.7%)	19 (19%)	0.417
Hepatic telangiectasia	58 (40.8%)	27 (27%)	0.182
FNH/NRH	10 (7%)	5 (5%)	0.827
Tortuous or enlarged hepatic artery	30 (21.1%)	13 (13%)	0.346
*Other intrabdominal involvement at abdominal CT*	46 (32.4%)	36 (36%)	0.639
Pancreatic	27 (19%)	19 (19%)	0.514
Splenic	13 (9.1%)	10 (10%)	0.912
Renal	6 (4.2%)	6 (6%)	0.675
Arterial	1 (0.7%)	2 (2%)	0.582
***Pulmonary assessment***			
*Pulmonary Manifestations*	14 (9.9%)	9 (9%)	0.822
Dyspnea	7 (4.9%)	2 (2%)	0.313
Respiratory failure	5 (3.5%)	1 (1%)	0.405
Hemoptysis	0	2 (2%)	0.170
Ischemic stroke	6 (4.2%)	5 (5%)	0.765
Abscess	4 (2.8%)	2 (2%)	1.000
*TTE*	139 (97.9%)	97 (97%)	0.693
R/L shunt contrast TTE	99 (69.7%)	62 (62%)	0.128
Shunt ≥2	53 (37.3%)	24 (24%)	0.022
sPAP at TTE, mmHg (mean ± SD)	32.92 ± 10.21	31.28 ± 10.07	0.387
sPAP> 40 mmHg	15 (10.5%)	5 (5%)	0.164
*Thoracic CT*	57 (40.1%)	24 (24%)	0.007
Pulmonary AVMs	50 (35.2%)	23 (23%)	0.036
***CNS assessment***			
*CNS manifestations*	3 (2.1%)	1 (1%)	0.644
Cerebral haemorrhage	1 (0.7%)	1 (1%)	1.000
Headache	2 (1.4%)	0	0.513
*CNS imaging study*	49 (34.5%)	42 (42%)	0.257
Pathological vascular imaging	7 (4.9%)	5 (5%)	0.706
**Gastrointestinal assessment**			
Suspected and studied	34 (23.9%)	24 (24%)	0.992
GI telangiectasia	29 (20.4%)	23 (23%)	0.223
Location of telangiectasia:			
Stomach	25 (17.6%)	17 (17%)	0.903
Duodenum	14 (9.8%)	21 (21%)	0.015
Colon	4 (2.8%)	6 (6%)	0.326
Ileum-Jejunum**	10 (7.1%)	7 (7%)	0.129

Abbreviations: *AVM* arteriovenous malformation, *CT* computed tomography, *sPAP* systolic pulmonary artery pressure, *FNH/NRH* focal nodular hyperplasia/nodular regenerative hyperplasia, *TTE* Transthoracic echocardiography, *R/L* Right-left, *CNS* central nervous system, *GI* gastrointestinal;

* Heart failure due to liver involvement

** Video capsule endoscopy was performed in 12 women and 11 men

### HHT severity during follow-up

Mean follow-up in women and men was 41.9 ± 25.8 and 40.4 ± 25.3 months, respectively. There were no significant differences between women and men in the baseline ESS (3.47 ± 2.19 vs 3.65 ± 2.12), mean ESS assessed at each visit during follow-up (2.21 ± 1.76 vs 2.52 ± 1.71) or in the number of patients with moderate (42.9% vs 37%) or severe ESS (6.3% vs 8%).

The Simple Clinical Scoring Index was higher in women (3.38 ± 1.20 vs 2.03 ± 1.24). More men were considered at low risk of harboring clinically significant liver disease than women (25.3% vs 61%), while all patients at high risk were women (4.9% vs 0%).

The HHT-score was similar in both groups (2.31 ± 1.06 vs 2.09 ± 0.88). No patients showed diffuse pulmonary AVMs, according to the HHT-score definition [25]. We have found no statistically significant differences between women and men regarding moderate (33.1% vs 29%) or severe (4.9% vs 1%) categories.

Women needed invasive treatments more often than men (28.2% vs 16%). Men needed attention to the ED more often than women (48% vs 28.2%), without statistically significant differences in severe anemia (hemoglobin level < 8 g/dL or RBC transfusion requirements), hospital admissions or mortality, or when considering any of these adverse outcomes (Table 3).

**Table 3:** Gender differences in HHT severity

	Female (n = 142)	Male (n = 100)	*P* value
***Follow-up, months (mean ± SD)***	41.9 ± 25.8	40.4 ± 25.3	0.647
***ESS***			
ESS at baseline (mean ± SD)	3.47 ± 2.19	3.65 ± 2.12	0.536
ESS during follow-up (mean ± SD)	2.21 ± 1.76	2.52 ± 1.71	0.242
ESS ≥ 4	61 (42.9%)	37 (37%)	0.379
ESS ≥ 7	9 (6.3%)	8 (8%)	0.604
***Simple Clinical Scoring Index***	3.38 ± 1.20	2.03 ± 1.24	< 0.001
Low	36 (25.3%)	61 (61%)	< 0.001
Intermediate	90 (63.4%)	31 (31%)	< 0.001
High	7 (4.9%)	0	0.043
***HHT-score***	2.31 ± 1.06	2.09 ± 0.88	0.083
Mild	88 (62%)	69 (69%)	0.259
Moderate	47 (33.1%)	29 (29%)	0.532
Severe	7 (4.9%)	1 (1%)	0.245
***Invasive treatment***			
Pulmonary embolization	34 (23.9%)	16 (16%)	0.126
Brain embolization	2 (1.4%)	0	0.470
Liver transplantation	2 (1.4%)	0	0.513
Young surgery	3 (2.1%)	0	0.270
Anyone of the above	40 (28.2%)	16 (16%)	0.027
***Adverse outcomes***			
Hemoglobin < 8 g/dL or RBC transfusion	49 (34.5%)	33 (33%)	0.578
RBC transfusion	37 (26.1%)	27 (27%)	0.870
ED visit	40 (28.2%)	48 (48%)	0.002
Hospital admission	27 (19%)	26 (26%)	0.196
Mortality	4 (2.8%)	4 (4%)	0.721
Anyone of the above	70 (49.3%)	53 (53%)	0.570

Abbreviations: *EES* Epistaxis Severity Score, *RBC* red blood cell, *ED* emergency department

### HHT severity according to HHT1 or HHT2 subtypes

HHT1 and HHT2 were documented in 155 (64%) out of the 242 patients included. Among them, 80 patients had HHT1 (49 women and 31 men) and 75, HHT2 (42 women and 33 men). There were no gender differences in epistaxis according to ESS in both HHT1 and HHT2. However, women showed higher mean of Simple Clinical Scoring Index when compared to men, either among HHT1 (2.97 ± 1.08 vs 1.62 ± 1.04) and HHT2 (3.64 ± 1.25 vs 1.93 ± 1.23) patients. Similarly, less women showed low risk of harboring clinically significant liver disease compared to men in both HHT1 (40.8% vs 67.8%) and HHT2 (14.3% vs 63.6%). Among HHT2 patients, women needed invasive treatment more often than men (16.7% vs 0%) but not in HHT1. There were no statistically significant differences in the HHT-score or adverse outcomes between women and men nor in HHT1 nor in HHT2 patients (Table 4).

**Table 4:** Gender differences in HHT severity according to HHT1 or HHT2 subtypes

	HHT 1 (*n* = 80)		*P* value	HHT2 (*n* = 75)		*P* value
	Female (*n* = 49)	Male (*n* = 31)		Female (*n* = 42)	Male (*n* = 33)	
***ESS***	2.82 ± 1.95	2.81 ± 1.95	0.977	3.67 ± 1.96	3.81 ± 1.80	0.744
ESS Control	2.12 ± 1.59	2.15 ± 1.89	0.937	2.25 ± 1.65	2.76 ± 1.50	0.238
ESS ≥ 4	16 (32.7%)	7 (22.6%)	0.332	20 (47.6%)	14 (42.4%)	0.669
ESS ≥ 7	1 (2%)	1 (3.2%)	1.000	2 (4.8%)	1 (3.1%)	1.000
***Simple Clinical Scoring Index***	2.97 ± 1.08	1.62 ± 1.04	< 0.001	3.64 ± 1.25	1.93 ± 1.23	< 0.001
Low	20 (40.8%)	21 (67.8%)	0.002	6 (14.3%)	21 (63.6%)	< 0.001
Intermediate	29 (59.2%)	6 (19.4%)	0.002	26 (61.9%)	10 (30.3%)	0.003
High	0	0		5 (11.9%)	0	0.058
***HHT-score***	2.24 ± 0.99	2.13 ± 0.86	0.599	2.35 ± 1.05	1.93 ± 0.82	0.058
Mild	30 (61.2%)	21 (67.7%)	0.429	28 (66.7%)	25 (75.8%)	0.391
Moderate	19 (38.8%)	9 (29%)	0.429	11 (26.2%)	8 (24.2%)	0.847
Severe	0	0		3 (7.1%)	0	0.251
***Invasive treatment,***						
Pulmonary embolization	17 (34.7%)	12 (38.7%)	0.716	5 (11.9%)	0	0.063
Brain embolization	1 (2%)	0	1.000	1 (2.4%)	0	1.000
Liver transplantation	0	0		1 (2.4%)	0	1.000
Young surgery	0	0		1 (2.4%)	0	1.000
Anyone of the above	18 (36.7%)	12 (38.7%)	0.859	7 (16.7%)	0	0.016
***Adverse outcomes,***						
Hemoglobin < 8 g/dL or RBC transfusion	8 (16.3%)	5 (16.1%)	1.000	16 (38.1%)	11 (33.3%)	0.452
ED visit	8 (16.3%)	11 (35.5%)	0.050	12 (28.6%)	16 (48.5%)	0.077
Hospital admission	6 (12.2%)	8 (25.8%)	0.120	8 (19%)	3 (9.1%)	0.328
Mortality	0	0		2 (4.8%)	1 (3%)	1.000
Anyone of the above	17 (34.7%)	13 (41.9%)	0.515	22 (52.4%)	16 (48.5%)	0.738

Abbreviations: *EES* Epistaxis Severity Score, *RBC* red blood cell, *ED* emergency department

## Discussion

To our knowledge, this is the first study after massive screening due to publication of international guidelines, assessing gender differences on clinical severity in HHT patients, either as a whole group or separetely by HHT1 and HHT2 [10]. In our series, hepatic manifestations were detected in 7.1% of women, while no man showed any of these manifestations. Women specifically showed heart failure signs and symptoms caused by liver involvement and hepatic arteriovenous shunt and biliar involvement more often than men [19, 28]. In fact, the Simple Clinical Scoring Index included female gender as a risk factor for developing clinically significant hepatic involvement [19]. This female predominance in prevalence and severity of liver involvement is in agreement with the four published series of HHT patients with liver transplantation, where between 83.3 and 92.8% of the patients were women [24, 32–34]. Moreover, 14 (60.8%) out of 23 of patients with high-output heart failure and all cases (*n* = 12) of isolated symptomatic biliar disease from the European Liver Transplant Registry were females, similar to our observation [24]. In fact, decompensated high output cardiac failure and biliary complications are the most frequent causes of liver transplantation [24, 32–34]. Similar to previous studies, among patients with genotype data available from our cohort, those at high risk of harboring clinically significant liver disease had *ACVRL1* mutation [6, 19]. A better understanding of liver involvement in HHT and improvements in selective screening and close monitoring from a hepatic standpoint, might contribute to early detection of those patients who will develop symptomatic liver disease and probably need liver transplantation, as female HHT2 population.

Although women also showed more prevalence of pulmonary AVMs, differently from hepatic involvement, they presented similar clinical pulmonary manifestations to men. Interestingly, some previous studies showed similar prevalence of pulmonary AVMs in women, although others showed that men are at greater risk of HHT related brain abscesses [16, 35–37]. In contrast to previous studies showing a higher prevalence of brain AVMs in women, we have found no gender differences [38–41]. However both in our study and the one by Letteboer et al., screening of brain AVMs was only performed according to either patient’s symptoms or family history, so the number of asymptomatic brain AVMs might be underestimated [42].

Regarding nasal or GI telangiectasia, neither ESS nor GI involvement (confirmed by endoscopic digestive study) did not show significant gender differences in our series. Surprisingly, our data revealed that men showed more telangiectasia in the duodenum. This finding should be confirmed in further studies. Our study also showed that women needed more invasive treatments during follow-up and that men visited the ED more often. However there were no gender differences regarding mortality or other adverse outcomes.

Though no differences between genders in terms of mortality were found, the reason for higher severity in liver involvement among women is still unknown and deserves special attention. The higher prevalence of women in our study, as it was in previous studies, does not justify this finding [12, 21, 22]. When assessing gender differences among patients with HHT1 or HHT2 separately, women had more severe liver involvement both in HHT1 and HHT2 patients. A higher impact in the angiogenesis process mediated by mutations in the signalling BMP9-Endoglin-ALK1-Smad hub among women, could be an explanation. However, this hypothesis does not justify the specific severity of liver involvement in women. The benefit of estrogenic therapy in HHT might suggest that a hormonal component could influence angiogenesis. Different studies have performed immunohistochemistry analyses of oestrogen and progesterone receptors expression among HHT patients with other vascular malformations [43–45]. These studies resulted in controversial results, and do not support a critical role of hormonal receptors in mucosa telangiectasia from HHT patients. Therefore, other unknown mechanisms should be involved in gender differences. New insights in the underlying mechanisms may help to gain a better understanding of HHT pathophisiology and angiogenesis process, and could help to develop new treatments or drug repositioning [46–49].

Our study has several strenghts and limitations that should be mentioned. A relatively low number of patients with severe clinical manifestations of hepatic and pulmonary involvement were included. Reasons for that might be that our HHT multidisciplinary referral unit attends all HHT patients and not only those more severely ill patients referred for invasive treatments. Moreover, our study was performed in a large real-world cohort of HHT patients with a consistent diagnosis by a specialized multidisciplinary team. Interestingly, all data were prospectively collected during long-term follow-up after HHT diagnosis, enhancing the ability to reflect the natural history of the disease and the wide spectrum of HHT vascular involvement. Finally, it should be noted that neither the Simple Clinical Scoring Index nor the general HHT score have been externaly validated [19, 25].

## Conclusions

In conclusion, the assessment of gender differences in overall HHT patients or separately by HHT1 and HHT2, revealed that women showed more severe liver involvement than men. Moreover, women had a higher prevalence of pulmonary AVMs and a higher need for invasive procedures, while men required ED assessment more frequently. No other gender differences were found in severity by using different reported scores, nor in mortality. These data might help physicians to better individualize follow-up according to gender and optimize therapeutic interventions in HHT patients.

## Data Availability

The datasets used and/or analysed during the current study are available from the corresponding author on reasonable request.

## Abbreviations

ACVRL1: activin A receptor type II-like 1
AF: Atrial fibrillation
AVMs: arteriovenous malformations
CI: Confidence intervals
CNS: Central nervous system
CT: tomography
ED: Emergency department
ENG: Endoglin
ENT: ear, nose and throat
ESS: Epistaxis severity score
GI: Gastrointestinal
HHT: Hereditary hemorrhagic telangiectasia
HR: Hazard ratios
LAA: left atrial appendage
MRI: Magnetic resonance
RBC: red blood cels
R-L: Right-left
SD: Standard deviations
STROBE: Strengthening the Reporting of Observational Studies in Epidemiology
TTE: Transthoracic echocardiography
